# Supplementary material for: Heat Stress-Tolerant Quantitative Trait Loci Identified Using Backcrossed Recombinant Inbred Lines Derived from Intra-Specifically Diverse Aegilops tauschii Accessions
Source: Plants (Basel). 2024 Jan 24;13(3):347. doi: 10.3390/plants13030347 (PMC10856904; doi:10.3390/plants13030347)
Supplement: Supplementary file 1 [file plants-13-00347-s001.zip › Figure S1(08.01.2024).pptx]

## Slide 1
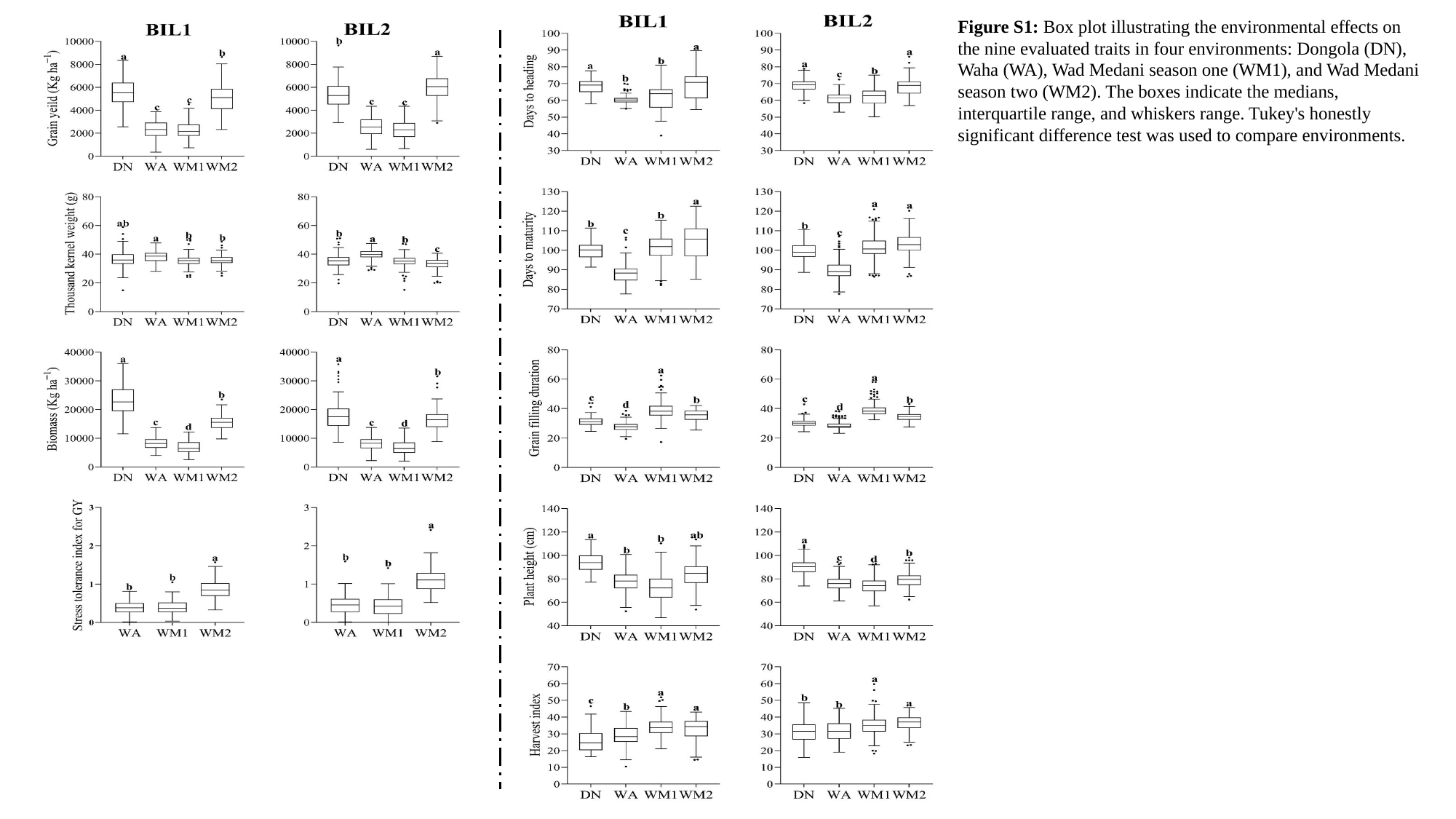

Figure S1: Box plot illustrating the environmental effects on the nine evaluated traits in four environments: Dongola (DN), Waha (WA), Wad Medani season one (WM1), and Wad Medani season two (WM2). The boxes indicate the medians, interquartile range, and whiskers range. Tukey's honestly significant difference test was used to compare environments.
